# Survey of Smallholder Dairy Cattle Farming System and Antibiotic Residues in Raw Cow's Milk in Bagamoyo District, Tanzania

**DOI:** 10.1002/vms3.71108

**Published:** 2026-07-29

**Authors:** Ridhiwan I. Ramadhani, Hezron Emmanuel Nonga

**Affiliations:** ^1^ Department of Veterinary Medicine and Public Health College of Veterinary Medicine and Biomedical Sciences Sokoine University of Agriculture Morogoro Tanzania

**Keywords:** drug withdrawal period, health risks, livestock husbandry practices, raw cow milk, veterinary drugs

## Abstract

**Background:**

Smallholder dairy farmers in Tanzania face multiple challenges, particularly livestock diseases, which drive the imprudent use of veterinary medicines most notably antimicrobials thereby contributing to antimicrobial resistance.

**Objectives:**

A cross‐sectional study was conducted in Bagamoyo District to examine smallholder dairy cattle production systems, health challenges, veterinary drug use and the presence of oxytetracycline (OTC) residues in raw cow milk.

**Methods:**

Structured questionnaires were administered to 110 farmers, and 50 mL of raw cow milk was collected from each for antimicrobial residue analysis. Screening for antimicrobial residues was performed using the microbial inhibition test, while identification and quantification of OTC residues were conducted using high‐performance liquid chromatography (HPLC). Data were summarised using descriptive statistics and analysed with Epi‐Info version 7.2.6.0.

**Results:**

Semi‐intensive feeding systems were most common (59.1%), and most farmers (91.8%) reported disease control through good animal management and hygiene (65.5%), pesticide use (63.4%), medicines (61.8%) and vaccination (56.4%). Vector‐borne diseases were prevalent, with East Coast Fever (67.3%), anaplasmosis (33.6%), and trypanosomiasis (21%) being the most frequently reported. Antimicrobials, particularly OTC, were widely used for treatment (63.6%) and were readily available from veterinary shops. Encouragingly, most farmers (96.4%) reported compliance with drug withdrawal periods, citing awareness of the health risks associated with consuming milk containing antimicrobial residues. The prevalence of antimicrobial residues in milk was 10%, with mean OTC concentrations of 785.6 ± 699.1 µg/L approximately eight times higher than the Codex Alimentarius Commission's maximum residue limit (MRL) of 100 µg/L.

**Conclusions:**

Such elevated levels pose a significant public health risk to consumers and compromise milk quality. Strengthening farmer education on good livestock husbandry, effective disease control and strict adherence to drug withdrawal periods is recommended to safeguard public health and improve milk safety standards.

## Graphical abstract

Smallholder dairy farmers in Tanzania face livestock disease challenges that drive imprudent antimicrobial use and fuel antimicrobial resistance (AMR). In Bagamoyo, poor husbandry and limited disease control result in high prevalence of vector‐borne infections, commonly treated with oxytetracycline (OTC). Despite reported compliance with withdrawal periods, mean OTC residues of 785.6 ± 699.1 µg/L were detected in milk. Such residues accelerate AMR, posing global health risks. Farmer education, legislative enforcement and strict adherence to withdrawal periods are critical interventions.

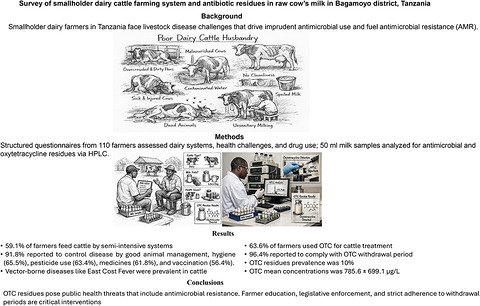

## 1 Introduction

Cattle production in Tanzania is dominated by indigenous breeds, particularly the Tanzania Shorthorn Zebu (TSHZ), which are raised by pastoralists and agro‐pastoralists under traditional systems reliant on extensive grazing (TLMP 2017; de Glanville et al. 2020). Indigenous breeds account for more than 95% of the national cattle population, estimated at 40.6 million (TLMP 2017; MLF 2026). Under the extensive systems, livestock keepers rarely implement preventive measures such as good husbandry practices, vaccination, vector control, or biosecurity interventions (Wilson 2003; TLMP 2017; de Glanville et al. 2020). Consequently, infectious diseases remain endemic, causing substantial annual losses to farmers, who often resort to veterinary drugs for disease prevention and control.

The dairy cattle population is estimated at 1.6 million, comprising crossbreeds of Friesian, Ayrshire, Jersey, and TSHZ (TLMP 2017; Ngou, 2026; MLF 2026). These animals are predominantly kept by smallholder farmers, with mean herd sizes of approximately four cattle, ranging from 1 to 12. Production systems include zero‐grazing, semi‐intensive, and, to a lesser extent, extensive grazing (TLMP 2017; Ngou, 2026; MLF 2026). A limited number of dairy ranches are owned by government institutions, non‐governmental organisations, religious bodies, farmer associations, and private individuals (TLMP 2017; Ngou, 2026; MLF 2026). Preventive measures are relatively better implemented in dairy farms; however, health challenges persist due to the susceptibility of exotic and crossbred cattle to infectious diseases, largely stemming from their limited genetic adaptation to tropical disease pressures (Wilson 2003; TLMP 2017; de Glanville et al. 2020).

Consistent with the cattle population structure, more than 80% of Tanzania's milk originates from indigenous cattle raised in rural areas (TLMP 2017; Lugamara et al. 2023). Annual milk production is estimated at 4.2 billion litres; however, over 90% is consumed in unprocessed form, as it is supplied through informal markets operated by smallholder producers (Lugamara et al. 2023; MLF 2026). Post‐harvest losses are substantial, ranging between 16% and 25% of total production, and are attributed to factors such as poor livestock husbandry, inadequate hygienic practices along the value chain, absence of cold chain infrastructure and market constraints (Lugamara 2024). National per capita milk consumption stands at 68.1 L, which is considerably lower than the 200 L per capita per year recommended by FAO standards (TDB 2020).

Cow milk produced in Tanzania is susceptible to contamination with antimicrobial residues due to widespread use of these drugs (Katakweba et al. 2012). Each year, Tanzania administers more than 1.6 million kilograms of antimicrobials to livestock (Sangeda et al. 2021). Since most livestock keepers operate under traditional, minimally regulated systems, farmers often access and administer veterinary drugs without restriction (Katakweba et al. 2012; Mashauri et al. 2025). However, compliance with recommended drug withdrawal periods prior to harvesting animal‐derived food products is rarely observed. Bagamoyo District hosts an estimated cattle population exceeding 301,623, the majority of which are TSHZ (Pwani Region Investment Guide 2019; MLF 2026). Many households also keep improved dairy breeds, which, together with the Shorthorn Zebu, constitute major sources of milk supplied to Dar es Salaam City (Pwani Region Investment Guide 2019; MLF 2026). In addition to three raw milk collection centres located in Msata, Chalinze, and Lugoba, individual traders and hawkers from Bagamoyo supply milk daily to the city sold to processing industries, designated collection points, or directly to households, street kiosks, and small restaurants (Pwani Region Investment Guide 2019). As in many parts of Tanzania, dairy farming in Bagamoyo suffers from poor husbandry practices and a high prevalence of livestock diseases, necessitating frequent use of veterinary drugs, particularly antimicrobials. There is also limited surveillance systems for antimicrobial residues, awareness, inadequate regulations, and knowledge, attitude, and practices leading to deposition of antimicrobials in milk (Mdegela et al. 2021). Previous studies in other regions of Tanzania have reported antimicrobial residues in cow milk at prevalences ranging from 1.7% to 68.3%, with concentrations between 10.4 µg/L and 134.9 µg/L (Kurwijila et al. 2006; Karimuribo et al. 2005; Gwandu et al. 2018; Kimera et al. 2015; Mashauri et al. 2025).

Despite Bagamoyo's role as a milk hub for Dar es Salaam City, no study has examined smallholder dairy cattle production systems or the status of antimicrobial residues in raw cow milk. This study therefore investigated dairy farming practices, health challenges, and the use and availability of veterinary drugs. It further assessed antimicrobial residue levels in raw cow milk and compared them with the maximum residue limits (MRLs) recommended by the Codex Alimentarius Commission (CAC). The findings provide valuable evidence for stakeholders across the milk value chain and for government authorities responsible for livestock and veterinary drug regulation.

## 2 Materials and Methods

### 2.1 Study Area and Population

**FIGURE 1:**
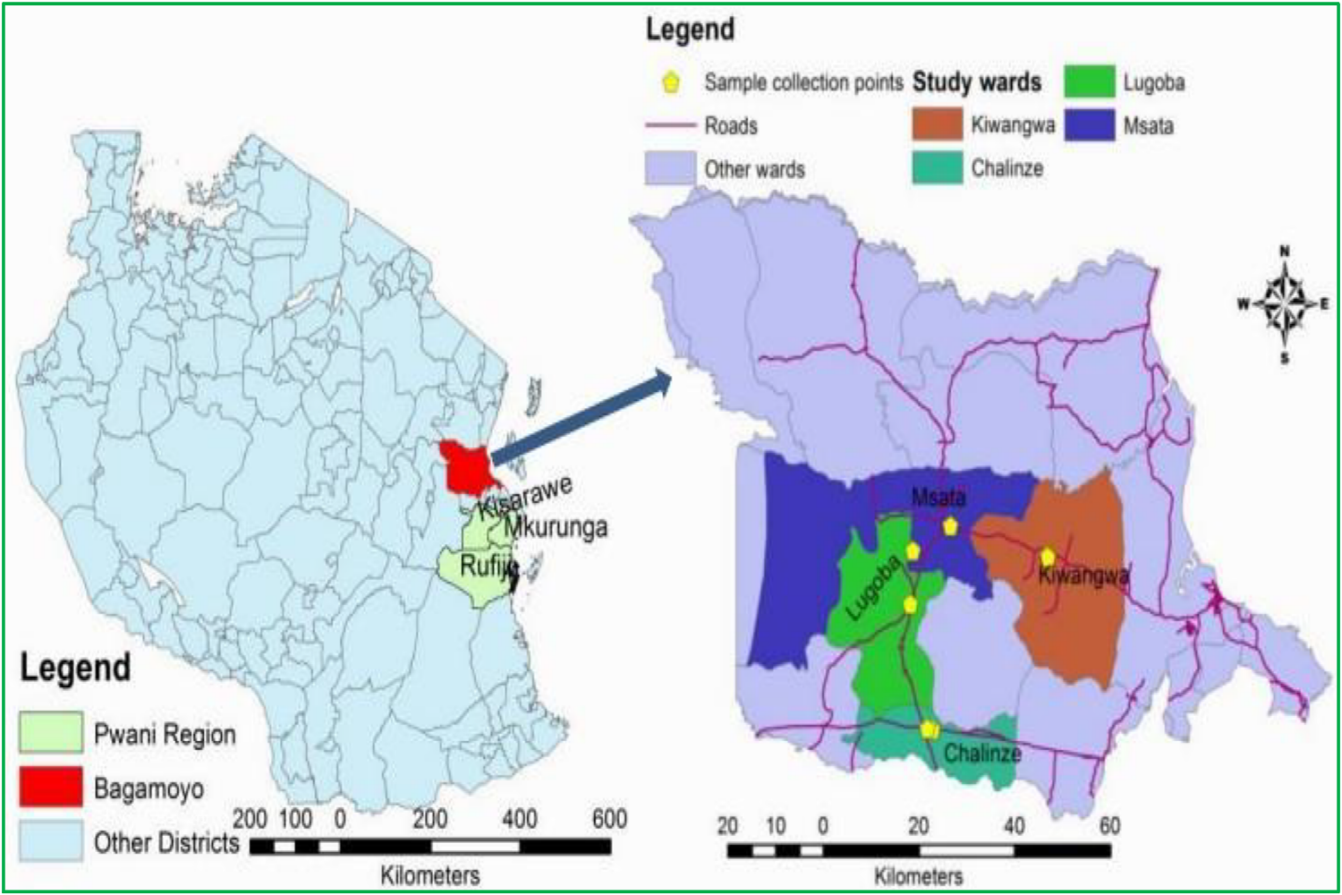
A map of Bagamoyo District showing study wards and sample collection points.

Bagamoyo is one of the seven districts of Pwani Region with two district council authorities, Bagamoyo with human population of 205,478 and Chalinze 316,759 (Figure 1). Administratively, Bagamoyo District Council has 11 wards while Chalinze has 15 (URT 2022). The rainfall pattern in the district is characterised by two rainfall seasons; long rainy season is in March to May and a short rainy season in October to December. The annual rainfall ranges from 800 mm to 1000 mm and the average temperature is 28°C. Bagamoyo District was selected for study because it has accessible raw milk collection centres of Msata, Chalinze and Lugoba (Figure 1) which ultimately supply to Dar es Salaam City.

The target study population was smallholder dairy farmers from the study wards. The small‐scale dairy production mostly keeps cross breeds of dairy cattle (Friesian, Ayrshire and Jersey) and the feeding system is semi‐intensive, zero (in‐door) grazing and sometimes extensive grazing. Lactating cows are supplemented with concentrates during milking time in the morning and evening. Routine diseases control mostly involve application of acaricides and occasionally vaccination.

### 2.2 Study Design, Selection Criteria and Sample Size

A cross‐sectional study design was employed that involved questionnaire administration and milk sample collection. The study wards were purposively selected based on its farmers supplying milk to the three collection centres. The selected wards that supply milk to Msata collection centre were Msata, Kiwangwa and Fukayosi, while Lugoba collection centre is supplied by farmers from Lugoba and Msoga wards. Additionally, Chalinze collection centre is served with milk by farmers from Bwilingu and Pera wards.

The sample size of 110 smallholder dairy farmers was calculated following the method described by Thrusfield (2005), using a 95% confidence interval (CI), 5% precision and expected prevalence of antimicrobial residues of 7% reported in Dar es Salaam, Tanzania (Kivaria et al. 2006). The formula used was:

$$n=\frac{Z_{\alpha /2}^{2}pq}{d^{2}}$$

where *n* is the estimated sample size; *Z* is the standard normal deviate at the 95% CI, corresponding to the level of significance (*Z* = 1.96); *p* is the expected antimicrobial prevalence of 7%; and *d* is the accepted margin of error (0.05). The final sample comprised 110 smallholder dairy farmers, including 41 from Msata, 19 from Lugoba and 50 from Chalinze.

### 2.3 Inclusion Criteria and Selection of Study Participants

The list of smallholder dairy farmers in the ward was obtained from the Ward Livestock Extension Officer. From the list, the smallholder dairy farmers inclusion criteria were having at least one lactating dairy cow during the study period, willingness to participate in the study, readiness to give information and accessibility of the place. The dairy farmers who met inclusion criteria were randomly selected to get representatives as per the calculated sample size. The selected farmers were categorised into clusters according to the specific milk collection centre they supply as clusters A, B and C for Msata, Lugoba and Chalinze milk collection centres, respectively.

### 2.4 Questionnaire Administration to Stallholder Dairy Farmers

Structured questionnaires were administered by face‐to‐face interviews with smallholder dairy farmers. The questionnaire consisted of three sections that addressed: (i) Demographic characteristics of smallholder dairy farmers, dairy cattle management systems and control of livestock diseases; (ii) Cattle health challenges, management of sick cattle, availability and use of veterinary drugs, and availability of veterinary services; and (iii) Veterinary drugs withdrawal and antimicrobial health risks.

Pretesting of questionnaires was done to test the clarity, sequence of questions and estimate the duration of each questionnaire. A total of 10 respondents from Mikese Ward in Morogoro District were interviewed and findings were used to improve the questionnaires. The revised questionnaire was translated and administered in Kiswahili, the national language of Tanzania. Further, the respondents were assured of anonymity, neutral and unbiased language was employed, the order of questions was randomised, and the questionnaires were kept concise. A structured questionnaire containing both closed‐ and open‐ended questions was used. Additionally, each participant was interviewed individually to encourage participation, and all interviews were conducted in private.

### 2.5 Milk Sample Collection and Handling

Raw cow milk sampling was conducted after completion of the questionnaire interviews. Milk samples from each farmer were collected directly from pooled milk storage containers using clean, sterile 100‐mL plastic bottles prior to being received and mixed in the main container at the centre. Approximately 50 mL of pooled raw milk was collected, labelled and stored in a cool box with ice packs during fieldwork. The samples were subsequently transported to the Microbiology Laboratory at Sokoine University of Agriculture for analysis and stored at −20°C until analysis.

### 2.6 Laboratory Analysis of Antimicrobial Residues

#### 2.6.1 Qualitative Analysis by Microbiological Inhibition Method

For test and interpretation of the results this study followed the general guidelines of Clinical and Laboratory Standards Institute (CLSI 2021). In microbiological inhibition test for drug residues agar well diffusion method was employed as previously described by Association of Official Analytical Chemists (AOAC 2000) and Syit (2011).

The culture media (Oxoid Ltd., Basingstoke, Hampshire, England, UK) were prepared in advance as described by the manufacturer and stored at 8°C refrigeration until use. *Bacillus subtilis* (ATCC 6633) was inoculated on Mueller–Hinton agar plates and incubated at incubated at 37°C for 24 h. Then two to three colonies of *B*. *subtilis* (ATCC 6633) were picked using a sterile wire loop and introduced into 5 mL of normal sterile saline in universal bottles and mixed. The turbidity of the mixture was then compared with a 0.5 McFarland standard. Subsequently 0.1 mL of already prepared *B. subtilis* (ATCC 6633) suspension was dispensed onto Mueller–Hinton agar plates and spread by using a sterile glass spreader for confluent growth as described by Luangtongkum et al. (2007). Then four wells or holes with diameter of 10 mm were punched into the agar layer using sterile boring glass rod on each plate. The wells were at a distance of at least 30 mm from each other. A 10 µL of the test raw milk sample was pipetted in the holes/wells and after a pre‐diffusion period of about 1 h at room temperature, the plates were incubated at 37°C for 24 h. The tests were performed in duplicate and both positive and negative controls wells/holes were included in each plate. Positive control was 10 µL of 5% oxytetracycline (OTC; Alfavet Animal Health Care, UK) and procaine penicillin dihydrostreptomycin (Norbrook Laboratories Ltd, UK) mixed with 1 mL of drug residues free UHT milk (Brookside Dairy Limited, Kenya). Negative control was 10 µL drug residues free UHT milk (Brookside Dairy Limited, Kenya). After 24 h of incubation, the cultures were examined for bacteria growth, the diameters of inhibition zones were measured with slipping callipers. An inhibition zone of ≥ 2 mm was considered positive (Aila et al. 2009; Nonga et al. 2009).

#### 2.6.2 Quantification of OTC Residues in Milk by HPLC Technique

The positive detected milk samples were confirmed and quantified by using high‐performance liquid chromatography (HPLC)‐UV and the test was done according to (AOAC 995.04). The procedures were conducted as described by Ghidini et al. (2003), with modifications implemented at the Tanzania Food and Drug Authority (TFDA) Laboratory in Dar es Salaam, Tanzania. The analytical details of HPLC are shown in Supporting Information 1.

### 2.7 Data Analysis

Raw data from survey and laboratory analysis were entered and stored in MS‐Excel spread sheet and analysed to describe samples with MRLs above the recommended values. Descriptive statistics including percentages, frequencies, medians and means were used to describe and summarise data. The data were analysed using Epi‐Info version 7.2.6.0 (CDC, USA) and computed for strength of associations using chi‐square and Fischer's exact test at critical probability of *p* < 0.05 at 95% CI.

## 3 Results

### 3.1 Sociological Results

#### 3.1.1 Demographic Characteristics of the Study Population

Results on socio‐demographic characteristics of smallholder dairy farmers and cattle production system in Bagamoyo are shown in Table 1. A total of 110 smallholder dairy farmers were interviewed, majority were female, primary school leavers, with the age of 36–55 years. The predominant cattle feeding system was semi‐intensive (59.1%), and most farmers (91.8%) reported implementing measures to control livestock diseases on their farms. Seven livestock disease control methods were reported, with the most practiced being good animal management and hygiene (65.5%), pesticide use (63.4%), medicinal treatments (61.8%) and vaccination (56.4%).

**TABLE 1:** Socio‐demographic characteristics of smallholder dairy farmers and cattle production system in Bagamoyo District (*n* = 110).

Parameter	Category	Number of farmers	Percentage
Sex	Male	79	71.8
	Female	31	28.2
Age (years)	15–35	18	16.4
	36–55	60	54.6
	Above 55	32	29.1
Education	Non‐formal	9	8.2
	Primary	64	58.2
	Secondary	30	27.3
	College	7	6.4
Area of residence	Chalinze	50	45.5
	Lugoba	19	17.3
	Msata	41	37.3
Feeding system	Semi‐intensive system	65	59.1
	Zero system	24	21.8
	Extensive grazing	21	19.1
Routine diseases control in cattle	Yes	101	91.8
	No	9	8.2
Type of diseases control in cattle	Good animal management including house hygiene	72	65.5
	Use of pesticides	70	63.4
	Use of medicines like antimicrobials and herbs	68	61.8
	Vaccination	62	56.4
	Routine of antitrypanosomes	45	40.9
	Routine deworming	35	31.8
	Washing of udder	18	16.4

#### 3.1.2 Commonly Reported Animal Diseases Affecting Dairy Cattle

**FIGURE 2:**
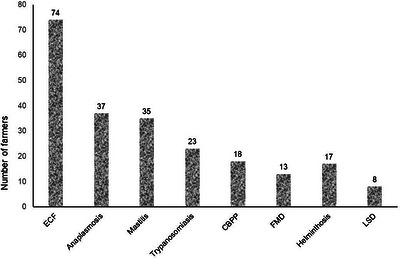
Eight cattle diseases reported by smallholder dairy farmers in Bagamoyo District. CBPP, contagious bovine pleuropneumonia; ECF, East Coast Fever; FMD, foot and mouth diseases; LSD, lumpy skin disease.

The small‐scale dairy farmers reported eight cattle diseases as shown in Figure 2. Tick‐borne diseases particularly East Coast Fever (ECF; 67.3%, *n* = 74) and anaplasmosis (33.6%) were reported by majority of the farmers.

#### 3.1.3 Use of Veterinary Drugs in Dairy Cattle

Table 2 summarises the veterinary drugs used in cattle as were mentioned by smallholder dairy farmers in Bagamoyo. A total of 11 drugs were mentioned dominated by antibiotics/antimicrobials (7/11), the other groups were trypanocides (2/11) and anthelmintics (2/11). OTC was reported by 65.5% of the farmers followed by Samorin (Isometamidium chloride; 47.2%).

**TABLE 2:** List of veterinary drugs used in cattle mentioned by smallholder dairy farmers in Bagamoyo.

Veterinary drug	Group	Number of farmers	Percent
Oxytetracycline	Antibiotic	72	65.5
Samorin (Isometamidium chloride)	Trypanocide	52	47.2
Mastitis infusion tubes	Antibiotic	48	43.6
Sulphonamides	Antimicrobial	43	39.1
OTC wound spray	Antibiotic	35	31.8
Penicillin and dihydrostreptomycin	Antibiotic	32	29.1
Albendazole	Anthelmintic	32	29.1
Levamisole	Anthelmintic	24	21.8
Berenil (Diminazene aceturate)	Trypanocide	21	19.1
Gentamycin drugs	Antibiotic	6	5.5
Chlortetracycline pessaries	Antibiotic	5	4.5

#### 3.1.4 Management of Sick Cattle, Veterinary Drug Availability and Drugs Withdrawal Period in Bagamoyo

Information on sick cattle management, availability of veterinary drugs and drugs withdrawal period are shown in Table 3. It was established that sick cattle were mostly (69.1%) attended by livestock experts who were easily accessible, the veterinary drugs were sourced from veterinary shops (49.1%). Furthermore, most respondents (67.3%) reported having never attended any training on the identification and management of sick cattle. Farmers reported using antimicrobials in cattle primarily for treatment and indicated awareness of drug withdrawal periods (96.4%). Most farmers (91.8%) reported complying with drug withdrawal periods and acknowledged the health risks associated with consuming cow milk containing antimicrobial residues (91.8%).

**TABLE 3:** Management of sick cattle, veterinary drug use and withdrawal periods in Bagamoyo.

Parameter	Category	Number of farmers	Percentage
Who treats sick cattle	Livestock experts	76	69.1
	A farmer	22	20.0
	Both farmers and livestock experts	12	10.9
Source of veterinary drugs	Veterinary shops	54	49.1
	Livestock experts	53	48.2
	Livestock markets	3	2.7
Availability and accessibility of livestock services	Timely, when needed	89	80.9
	Difficult to get	21	19.1
Training in identification and management of sick cattle	No training	74	67.3
	Yes, trained by veterinary drug sellers, livestock experts and from media	36	32.7
Use of antimicrobials in cattle	Yes	94	85.5
	No	16	14.5
Purpose of antimicrobial uses (*n* = 54)	Treatment	44	81.5
	Treatment and prophylaxis	10	18.5
Aware of drugs withdrawal period	Aware	106	96.4
	Not aware	4	3.6
Compliance with withdrawal period	Comply	101	91.8
	Not comply	9	8.2
Awareness of health risks associated with consumption of cow milk with antimicrobial residues	Aware	101	91.8
	Not aware	4	3.6
	Did not know anything	5	4.6

### 3.2 Laboratory Results

#### 3.2.1 Qualitative Screening for Antimicrobial Residues

A total of 110 raw cow milk samples were collected, comprising 41 (37.3%) from the Msata collection centre, 19 (17.3%) from Lugoba, and 50 (45.5%) from Chalinze, as shown in Table 4. Of the 110 samples screened, 11 (10%) exhibited inhibition zones suggestive of antimicrobial residues. The Chalinze collection centre recorded the highest number of positive samples (5.5%, *n* = 6), compared with Msata (4.5%, *n* = 5); however, no significant difference was observed between the two centres (*p* = 0.9773; OR: 0.9818; 95% CI, 0.2768–3.4819). All samples from Lugoba showed no detectable antimicrobial residues (Table 4).

**TABLE 4:** Prevalence of antimicrobial residues in raw cow milk in Bagamoyo District.

Milk collection centre	No. of sample	No. positive	Percentage contaminations
Chalinze	50	6	12.0
Msata	41	5	12.2
Lugoba	19	0	0.0
Total	110	11	10.0

#### 3.2.2 Quantitative Analysis of OTC in Raw Milk Samples

The 11 milk samples that exhibited inhibition zones were further analysed using HPLC to confirm the presence of antimicrobial residues, with results presented in Table 5. All the samples contained detectable levels of OTC residues, exceeding the CAC and European Union (EU) MRL of 100 µg/L. The mean OTC concentration was 785.6 ± 699.1 µg/L, with values ranging from 372.5 to 2841.1 µg/L. Notably, the average OTC concentration in milk from Bagamoyo District was nearly eight times higher than the recommended Codex MRL. Furthermore, all positive samples surpassed the Codex threshold. At the Chalinze collection centre, six samples (54.6%) tested positive for OTC residues (Table 5).

**TABLE 5:** Oxytetracycline levels in raw cow milk samples from Bagamoyo District.

Location	Sample code	OTC residues (µg/L)
Chalinze	C3	395.3
Chalinze	C5	2841.1
Chalinze	C7	678.6
Chalinze	C8	483.0
Chalinze	C9	548.1
Chalinze	C10	925.4
Msata	A4	553.7
Msata	A5	597.8
Msata	A11	526.1
Msata	A12	719.9
Msata	A13	372.5

## 4 Discussion

This study examined smallholder dairy cattle production systems, health challenges, the use of veterinary drugs and the presence of OTC residues in raw cow milk in Bagamoyo District. Overall, dairy cattle production was found to be managed under intensive and semi‐intensive systems, with disease control measures primarily involving good animal management and hygiene, pesticide use, medicinal treatments and vaccination. However, mastitis and vector‐borne diseases, in particular ECF were common. Different treatments were administered using various veterinary drugs, predominantly antimicrobials that were readily available from veterinary shops. Livestock experts attended to sick cattle, and farmers reported observing drug withdrawal periods; however, the prevalence of OTC residues in milk was 10%, with a mean concentration of 785.6 ± 699.1 µg/L. These findings underscore the urgent need to support smallholder farmers in adopting sound livestock husbandry practices for disease control, adhering to antimicrobial stewardship principles, and observing drug withdrawal periods to prevent residues that pose serious public health risks, including antimicrobial resistance.

The study revealed that most smallholder dairy farmers employed intensive or semi‐intensive production systems, in which pasture was provided indoors alongside concentrate supplementation. At times, animals were grazed outdoors around the homestead for limited hours and supplied with water indoors. This feeding system is commonly practiced for dairy cattle in various parts of Tanzania (Ngou, 2026). When properly implemented, this form of husbandry can reduce health problems and enhance productivity. Farmers reported employing various disease control measures, particularly good animal management practices, improved hygiene in animal housing, the use of pesticides and medicines, and vaccination. Nevertheless, they frequently encountered several cattle diseases, most notably vector‐borne infections such as ECF, anaplasmosis and trypanosomiasis. Mastitis and contagious bovine pleuropneumonia (CBPP) were also commonly reported. Livestock diseases continue to undermine cattle health and productivity, contributing to an estimated 20% annual loss in livestock production (Cervantes‐Godoy and Dewbre 2020). Vector‐borne diseases, CBPP, and mastitis remain among the most prevalent cattle diseases in Tanzania (Chengula et al. 2013; Ndonde et al. 2025). It is therefore imperative that livestock extension officers provide farmers with guidance on improved husbandry practices and effective disease control strategies, including vaccination, pesticide application and biosecurity measures.

Furthermore, the use of veterinary drugs for both treatment and disease prevention was widespread. A total of 11 veterinary drugs, predominantly antimicrobials, were reported to be in use and were readily accessed through veterinary shops and livestock experts, as documented in previous studies (Jaime et al. 2022; Katakweba et al. 2012; Mdegela et al. 2021). In addition to therapeutic purposes, the ease of access may contribute to indiscriminate use, which poses several challenges (Katakweba et al. 2012; Mdegela et al. 2021; Sangeda et al. 2021). Although existing legislation strictly regulates access to and use of veterinary drugs, enforcement remains weak (Mdegela et al. 2021; Sangeda et al. 2021).

The current study determined the prevalence of OTC residues in milk to be 10%. Other studies in Tanzania have reported both higher and lower prevalence rates compared to those observed in the present study (Kurwijila et al. 2006; Karimuribo et al. 2005; Gwandu et al. 2018; Kimera et al. 2015; Mashauri et al. 2025). Elsewhere in African countries reported different prevalence of antimicrobial residues in milk (Wambua 2016; Kosgey et al. 2018; Zulu et al. 2025; Nanteza et al. 2026). Such variation may reflect differences in laboratory methodologies and the extent of non‐compliance with drug withdrawal periods.

According to questionnaire responses, most farmers reported awareness of drug withdrawal periods and claimed adherence, citing knowledge of the health risks associated with consuming milk containing antibiotic residues. However, these findings suggest that some farmers may have provided socially desirable responses while continuing to sell milk from cattle under antibiotic treatment. This may be due to the tendency of some farmers to over‐report positive behaviours and under‐report negative to please the researchers, perceived societal norms, portray themselves favourably and avoid judgement (Wambua 2016; Zulu et al. 2025). Another possible explanation for the apparent mismatch between farmers’ reported high awareness of drug withdrawal periods, their claimed compliance, and the observed prevalence and elevated concentrations of antimicrobials in milk is that many farmers did not actually understand the meaning of withdrawal periods. However, the non‐compliance may be driven by economic necessity. For many smallholder farmers, discarding milk during the withdrawal period represents a total loss of daily cash flow which may be overcome by provision of financial incentives and strict regulatory testing at collection centres (Mashauri et al. 2025; Zulu et al. 2025). It was further established that nearly 31% of farmers admitted to treating their cattle without veterinary supervision, despite lacking adequate knowledge of antimicrobial dosages, routes of administration, and withdrawal periods factors that substantially increase the risk of antimicrobial residues in milk (Wambua 2016; Mashauri et al. 2025; Zulu et al. 2025).

Surprisingly, all the 11 positive samples had detectable levels of OTC residues at mean concentration of 785.6 ± 699.1 µg/L; which is eight times above the MRL of 100 µg/L recommended standards set by CAC. Food and Agricultural Organization, World Health Organization (FAO/WHO) and the EU‐CAC set such MRL for tetracycline, OTC and or chlortetracycline in milk considering the health effects caused by this group of antibiotics (Applegren et al. 1999; EU 2009; CAC 2023). Previous studies in Tanzania reported OTC residues concentrations in raw cow milk to range from 10.4 ug/L to 134.9 µg/L (Kimambo et al. 2026; Mhozya and Nonga 2026; Mashauri et al. 2025). Elsewhere, Aduda (2022) reported OTC residue concentrations in Kenya ranging between 15 µg/L and 17 µg/L, whereas in Uganda, levels of 137.6–325.21 µg/L were documented (Nanteza et al. 2026; Hebbal et al. 2020). In Nigeria, Malgwi et al. (2023) reported concentrations of 36 µg/L. Therefore, the detected high levels of OTC residues pose a threat to milk consumers and immediate intervention measures are recommended (Garcia et al. 2020; Virto et al. 2022).

### 4.1 Limitation of the Study

This study was a cross‐sectional design that involved questionnaire administration and collection of milk samples for antibiotic analysis in Bagamoyo only while Tanzania has 184 district councils. Although the results are relevant and type of dairy cattle farming in Bagamoyo represents the entire country, generalisation of results may be limited because of differences in climatic conditions and farming systems. Interviewing smallholder dairy farmers at milk collection centre levels and subsequently collection of pooled milk samples may have limited a lot of information which otherwise would be gathered at farm level. Where resources would be allowed, all the 110 raw cow milk samples would have been analysed for different ranges of commonly used antimicrobials in veterinary practices.

### 4.2 Conclusion

Smallholder dairy farmers in Bagamoyo practice intensive and semi‐intensive cattle production system, their cattle suffer from different diseases which necessitate uses of veterinary drugs especially antimicrobials. Although the majority of farmers reported observing drug withdrawal periods, laboratory analysis revealed a 11% prevalence of OTC residues in milk, with a mean concentration of 785.6 ± 699.1 µg/L. This study has provided baseline information on antimicrobial uses, withdrawal period, prevalence and concentrations of antimicrobial residues in milk. The observed high levels necessitate for an urgent intervention strategy by the Tanzania Bureau of Standards (TBS) and Tanzania Dairy Board (TDB) to strengthen monitoring and surveillance of antimicrobial residues in milk and other animal source foods to assure food safety and public health. It is insisted that Veterinary Council of Tanzania (VCT) and Tanzania Medicines and Medical Devices Authority (TMDA) and VCT should enforce legislations to combat haphazard sales and uses of veterinary medicines. Since routine screening for antibiotic residues is not conducted in milk collection centres, microbial inhibition tests may offer a cost‐effective and user‐friendly alternative. Immediate interventions are needed to improve smallholder farming practices, particularly in disease control, farmer education on antimicrobial stewardship and strict adherence to drug withdrawal periods, to prevent residues in milk that pose serious health risks. Furthermore, a coordinated One Health approach is essential to safeguard cattle health, ensure milk safety, protect consumer well‐being, and preserve the effectiveness of antimicrobials.

## Author Contributions

**Ridhiwan I. Ramadhani**: conceptualisation, investigation, writing – original draft, methodology, formal analysis, data curation. **Hezron Emmanuel Nonga**: conceptualisation, funding acquisition, methodology, writing – review and editing, formal analysis, project administration, supervision.

## Funding

The authors have nothing to report.

## Supporting information




**Supporting Information File 1**: vms371108‐supp‐0001‐SuppMat.doc

## Data Availability

The data that support the findings of this study are available upon request from the corresponding author. The data are not publicly available due to privacy or ethical restrictions.
