# Supplementary material for: Survey of Smallholder Dairy Cattle Farming System and Antibiotic Residues in Raw Cow's Milk in Bagamoyo District, Tanzania
Source: Vet Med Sci. 2026 Jul 29;12(5):e71108. doi: 10.1002/vms3.71108 (PMC13418862; doi:10.1002/vms3.71108)
Supplement: Supplementary file 1 — Supporting Information File 1: vms371108‐supp‐0001‐SuppMat.doc [file VMS3-12-e71108-s001.doc]

**SUPPLEMENTARY MATERIALS**

**1.0 Analysis of antimicrobial residues in milk by chromatographic method**

## **1.1 Equipment and Apparatus**

Analysis was carried out on a HPLC Model LC-20AT Prominence Liquid Chromatography series, serial No.C213346 015664, Shimadzu Corporation, Japan), equipped with constant flow pump, degasser (DGU 20A_5_ Serial L-20244 07326), column oven (CTO-10A VP Serial C 210447 06967), auto sampler (Optimas- Spark) and computer software. Separations were conducted by Zorbax Eclipse XDB-Analytical C18 (150x 4.6 mm, 5μm) Column Agilent Technologies-USA and analyte were detected by PDA-Detector with variable wavelength UV detector (SPD-20A), CBM- 20A Prominence serial No L 202347 09807 Shimadzu Corporation Japan. Other equipment used were included centrifugation during sample preparation and extraction was performed with a Rotofix 32A ®(Zentafugen)-Centrifuge made from Germany; Sonicator &Degasser used to degas mobile phase- Branson ® 3210E-DTH, UK; Nitrogen drier used to dry sample during extraction process-Thermo Scientific heating module ®No. 8824; PH Meter measuring +- 0.05 unit, - Jenway ® 3540 model-UK; Analytical balance-Readability 0.0001g – Ohaus® corporation-USA; Mechanical shaker/Vortex mixer- SFI, Sturt Scientific- UK; Clean-up Solid phase extraction (SPE) Cartridges-Varian Bond Elut Sax, USA; and sterile membrane filter- 0.45 μm pore size, Cat no. 7141114supplied from Japan

## **1.2 Chemicals and reagents**

Methanol-HPLC grade, acetonitrile- HPLC grade and Water- HPLC grade were supplied by Fishers Scientific Limited- UK; Disodium hydrogen phosphate dihydrate, oxalic acid-dihydrate, citric acid- monohydrate, Sodium phosphate-dibasic anhydrous, Ammonium hydroxide was purchased from Fishers Scientific Limited, UK; Ammonium acetate (Analytical grade) was supplied by Scharlau chemie S.A; EDTA, trifluoroacetic acid was purchased from Ps Park Scientific Limited- UK; Distilled and deionized water was obtained from TFDA Laboratory; and OTC CRS was supplied by the council of Europe (batch/lot no 4 a)

## **2. Sample preparation and extraction**

**2.1 Sample extraction**

Homogenized, previously frozen raw milk samples (5.0 ±0.1ml test milk), showing no signs of souring or curdling, were pipetted into 50 ml polypropylene centrifuge tube and centrifuged for 15 minutes at 250 rpm at approximately 10ºC to separate cream. The contents were mixed with 20 ml Mcllvaine/EDTA solution (S.D. Fine – Chem Ltd, Mumbai 400025, India) and shaken for 10 minutes on flat bed shaker or vortex mixer at high speed. The content of the tubes was then centrifuged for 20 minutes at a speed of 4000 rpm at approximately 15ºC. The supernatant was filtered through GF/B filter paper after moistening with Mcllvaine buffer-EDTA solution (S.D. Fine – Chem Ltd, Mumbai 400025, India).

## **2.2 Sample clean up by solid phase extraction (SPE)**

Sold phase extraction (SPE) cartridges were attached to an SPE vacuum manifold connected to 75 ml reservoir to each cartridge. The Bond Elut-Sax C18 cartridges were first conditioned with 20 ml methanol followed by 20 ml of HPLC-grade water at 1.5 - 2.5 ml/minute, vacuum was applied gently, and eluates were discarded. The final extract supernatant of test solution was then applied into C18 cartridge followed by drained cleaned and rinsed the cartridge with 20 ml HPLC- grade water and eluates were discarded. The cartridges were dried when water rinse was completed and continued to draw air through cartridge for approximately 2 minutes. The OTC residues test solution from the cartridges were eluted with 5.0 ml methanolic oxalic acid and filtered through 0.45 μm syringe filtration cartridges into LC autosampler vials and loaded into autosampler. Finally, 20 μl of the solution was injected into the HPLC-UV system. Samples were analyzed at 365 nm wavelength.

## **3.0 HPLC analysis for OTC residues**

Using 0.45 μm pore size micro syringe filter the test solution and the standards were filtered into the LC auto sampler vials and loaded into auto sampler. The analysis and quantification of the OTC residues in the extract was done using a HPLC (LC-20AT Model Shimadzu Prominence series -Japan) equipped with CBM-20A auto injector with sample cooler CTO-10AS VP and DGU- 20A_5_ on-line vacuum degassing solvent delivery unit, a constant flow pump and a variation wavelength UV detector set at 365 nm. The separation was done on Zorbax Eclipse XDB-Analytical C18 (150x 4.6 mm, 5 μm) column with Ammonium acetate- Acetonitrile solution (77:23) as the mobile phase by gradient mode, the mobile phase flowrate of 1.2 ml/min at room temperature and the sensitivity range was 0.08 ppm. The HPLC analysis was performed for 5 minutes in each sample.

To get the concentration of a given sample, reference standards of known concentrations were used to determine the concentration of the sample extract. For the determination of OTC residues, the OTC standard solutions at different concentrations of 12.5, 25, 50, 100 and 200 μg/l were injected in ascending order (Table 1). The extract from each sample was injected in duplicate to obtain average peak height of positive samples. The samples were positive for tetracycline if their retention time and peak corresponded to that of the reference standard. The retention time of the standard was 2.7 minutes as shown in Figure 2.

**Table 1. OTC Standards calibration curve**

| OTC reference standards (ug/l) | Area of the chromatography |
| --- | --- |
| 12.5 | 800.3 |
| 25.692 | 1473.3 |
| 47.465 | 2478 |
| 93.009 | 4853 |
| 198.561 | 10289 |


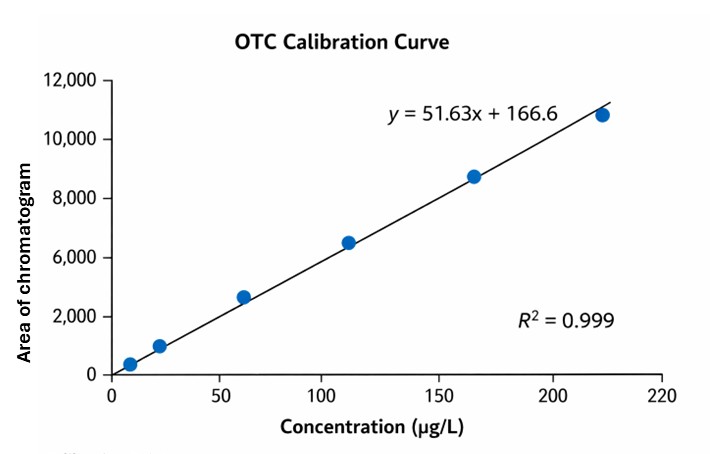


**Figure 2.** Linear calibration curve for oxytetracycline (OTC) standards (12.5–198.6 µg/L) with regression equation
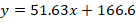
 and
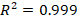
, demonstrating excellent linearity for quantitative analysis.

## **2.4 Method development for HPLC analysis of raw milk samples**

Prior to extraction of OTC in an unknown raw milk sample, the method was developed under laboratory conditions suitable for analysis using HPLC. The developed method was later used to determine OTC residues concentration of the raw milk sample in the present study.

**2.5 Preparation of OTC standard solutions**

Up to 180 mg of OTC standard solution was weighed and transferred into 100 ml amber volumetric flask, then was dissolved in methanol at room temperature. This OTC stock standard solution-1000 μg/ml was protected from light by using amber volumetric flask and stored at -20^o^C for three months and it was used to prepare the working OTC standard by serial dilutions.

The OTC intermediate stock solution-100 μg/ml was then prepared by diluting 10 ml of stock standard solution in 100 ml volumetric flask with methanol at room temperature and stored in -20^o^C. The intermediate stock solution was further diluted with methanol to get the OTC working standard solution 25 μg/ml. Then five OTC chromatographic standard solutions at 12.5, 25, 50, 100 and 200 μg/L concentrations were prepared using methanolic oxalic acid solution. Under gradient conditions, different standard concentrations of the OTC analytical standard solutions were injected in ascending order and the results were plotted automatically on the integrator. The machine was set to run for 5 minutes, and OTC was detected at 2.7 minutes as retention time with the peak height increasing with increase in concentrations.


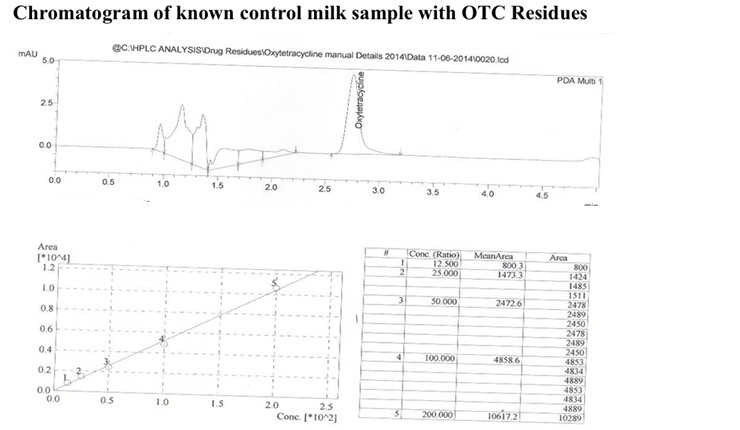


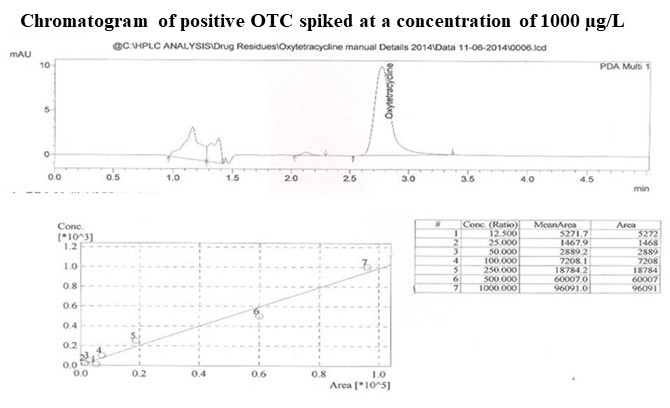


## **2.6 The positive and negative control samples**

A well-known negative and positive sample was used as quality control. Negative controls used was antimicrobial free UHT milk (Brookside Dairy) while positive controls were the UHT milk (Brookside Dairy) fortified (spiked) with a known quantity of OTC (Table 3). The negative control samples of duplicate UHT milk were treated the same way as the raw milk samples through undergone extraction, SPE clean-up and elusion with methanolic oxalic acid.

A total of 200 μl of the OTC standard working solution (25 μg/ml was pipetted into 5 ml volumetric flask and antimicrobial free UHT milk was added to the volume and mixed thoroughly by shaking for 10 seconds and left for 30 minutes before start extraction procedure with Mcllvaine buffer, solid phase extraction and elution from the cartridges. The control samples were run through the analytical column in a HPLC machine mobile phase conditioned with Ammonium acetate- Acetonitrile solution in a ratio of 77:23, respectively. The column temperature and flow rate were maintained as the case for the OTC analytical standard solutions. The retention time of the control samples was compared with those of the OTC analytical standard solution alone. During extraction, blank sample eluted from SPE cartridge was included to check for the analytical column efficiency.

## **2.7 LOD and LOQ of an analytical system**

Limit of detection (LOD) and Limit of quantification (LOQ) depend on the noise and drift of the detection equipment. Absolute detector LOD can be determined by injecting a sample directly into detector. It is often expressed at a minimum detectable level. However, LOD depends on the oxygen content of the mobile phase, the injection system, peak broadening on the column and temperature differences among system components. Taking these factors into account, the LOD was defined as 3 times the noise level and LOQ was defined as 10 times the noise level and in this study, it was determined by the analysis of spiked free from antimicrobial UHT milk (Brookside brand) sample with 10 μg/l. The recoveries of the antimicrobials were calculated at four different concentration levels of OTC Standards (50, 100, 500, 1000 μg/l) and on average the recovery percentage was 83% (Table 2).

## **Table 2.** Recovery and precision of OTC determined in spiked UHT milk sample (n=4).

| OTC Spiked level (μg/L) | Determined time | Recovery level (μg/L). | Mean recovery level (μg/L) | % recovery |
| --- | --- | --- | --- | --- |
| 50 | 1  2  3 | 28.269  26.934  26.934 | 27.379 | 54.8 |
| 100 | 1  2  3 | 66.024  67.522  72.918 | 68.821 | 68.8 |
| 500 | 1  2  3 | 532.166  552.881  589.700 | 558.249 | 111.6 |
| 1000 | 1  2  3 | 1007.021  950.356  - | 978.6885 | 97.9 |

Mean % Recovery = 83.3 and therefore extraction efficiency was 83.3%

## **3.0 Calculations**

The HPLC machine data output measured and recorded the peak area for OTC standard solutions and the test solutions. Using data from the OTC standard solutions, the best fit line of data was determined using the linear regression equation as follows; *y = mx +b* whereby: y = Sample peak area; x = OTC concentration in μg/l; m = slope and b = intercept of y. The correlation coefficient (R^2^) determined from the equation was 0.99636. The quantification of OTC residues of the sample analyte was determined by using the peak area of the analyte and values from the regression slope and intercept of the OTC standard solutions. Each OTC standard curve should be linear. Concentration of OTC residues in original raw milk was calculated directly since the dilution factor of injected test solution was 1 (eluted final volume in SPE cartridge). Care was taken with integration and therefore baselines were checked for each chromatogram determined by automated data systems.
